# Ginsenoside Rg3 Prolongs Survival of the Orthotopic Hepatocellular Carcinoma Model by Inducing Apoptosis and Inhibiting Angiogenesis

**DOI:** 10.1155/2019/3815786

**Published:** 2019-08-26

**Authors:** Shen Hu, Yan Zhu, Xiangyang Xia, Xiaobo Xu, Fen Chen, Xudong Miao, Xinmei Chen

**Affiliations:** ^1^Department of Obstetrics, The Second Affiliated Hospital of Zhejiang University School of Medicine, Hangzhou 310000, China; ^2^Department of Respiratory Hangzhou Shulan Hospital, Hangzhou 310009, China; ^3^Department of Ultrasound, The Second Affiliated Hospital of Zhejiang University School of Medicine, Hangzhou 310000, China; ^4^Department of Hepatobiliary and Pancreatic Surgery, Key Laboratory of Combined Multi-Organ Transplantation, Ministry of Public Health, The First Affiliated Hospital, School of Medicine, Zhejiang University, Hangzhou, 310003 Zhejiang Province, China; ^5^The Department of Orthopedics, The Second Affiliated Hospital, Zhejiang University, Hangzhou, Zhejiang 310003, China; ^6^The Department of Pharmacy, Shandong University of Traditional Chinese Medicine, Jinan, 250014 Shandong Province, China

## Abstract

**Aim:**

Microvessel density is a marker of tumor angiogenesis activity for development and metastasis. Our preliminary study showed that ginsenoside Rg3 (Rg3) induces apoptosis in hepatocellular carcinoma (HCC) *in vitro*. The aim of this study was to investigate the cross-link for apoptosis induction and antiangiogenesis effect of Rg3 on orthotopic HCC *in vivo*.

**Methods:**

The murine HCC cells Hep1-6 were implanted in the liver of mouse. With oral feeding of Rg3 (10 mg/kg once a day for 30 days), the quantitative analysis of apoptosis was performed by using pathology and a transmission electron microscope and microvessel density was quantitatively measured by immunohistochemical staining of the CD105 antibody. The mice treated with Rg3 (*n* = 10) were compared with the control (*n* = 10) using Kaplan-Meier analysis. Animal weight and tumor weight were measured to determine the toxicity of Rg3 and antitumor effect on an orthotopic HCC tumor model.

**Results:**

With oral feeding of Rg3 daily in the first 30 days on tumor implantation, Rg3 significantly decreased the orthotopic tumor growth and increased the survival of animals (*P* < 0.05). Rg3-treated mice showed a longer survival than the control (*P* < 0.05). Rg3 treatment induced apoptosis and inhibited angiogenesis. They contributed to the tumor shrinkage. Rg3 initialized the tumor apoptotic progress, which then weakened the tumor volume and its capability to produce the vascularized network for further growth of the tumor and remote metastasis.

**Conclusion:**

Rg3 inhibited the activation of microtumor vessel formation *in vivo* besides its apoptosis induction. Rg3 may be used as an adjuvant agent in the clinical HCC treatment regimen.

## 1. Introduction

Hepatocellular carcinoma (HCC) is one of the fifth most common cancers worldwide and the third most common cause of cancer death [1–3]. HCC is a highly vascularized tumor, and thus, the antiangiogenesis treatments such as arterializations and embolization have been applied; the overall clinical effect is not satisfying [4]. The metastasis and recurrence of hepatocellular carcinoma are still very challenging [5, 6].

HCC is especially highly prevalent in China, mainly attributed to the prevalence of hepatitis B virus (HBV) persistent infection and HBV-induced cirrhosis [7, 8]. The majority of HCC patients are already in advanced stage on the first diagnosis; on diagnosis, the majority of patients have already lose the opportunity for radical surgery or liver transplantation; the median survival is usually less than one year due to the absence of effective target medicine [8, 9]. Because HCC is generally originated from chronic hepatitis and many patients suffer from cirrhosis, the treatment is even more challenging than other malignancies. The underlying liver disease hinders the liver function and limits the application of the aggressive treatments. The effect and the toxicity are two sides that must be conjointly balanced.

Sorafenib is the only approved medicine, but it can only cure part of patients [10]. The local-regional ablation is recommended as the first-line treatment, but ablation currently works for the early stage HCC but fails for the advanced HCC cases [11]. Transarterial chemoembolization (TACE) is now accepted by many centers as a palliative treatment for HCC larger than 5 cm or multinodular lesions [4, 12]. It can increase the chemotherapeutic concentration in the tumor, but it can cause vessel obstruction and reduce hepatic ischemia. Even for the target therapy like Y90 microspheres, immune therapy has been tested but its long-term outcome is still uncertain for treating HCC by bioelectric ablation with microsecond pulsed electric fields (*μ*sPEFs) which is in the experimental period [13]. In fact, the therapeutic options in advanced HCC are still very limited, so the novel treatment with high target but low toxicity is in great need. Ginseng is a Chinese tradition herbal medicine; ginsenoside Rg3 is a chemical compound isolated from ginseng. Ginseng has a wide spectrum of pharmacological effects such as antiglucose tolerance, antioxidant status, and antioxidative stress in type 2 diabetes [14]; anti-inflammatory effects of ginsenoside Rg3 in A549 cells and human asthmatic lung tissue [15]; and induction of nitric oxide synthase of ginsenoside Rg3 to relax vessels [16]. Researchers have found that ginsenoside Rg3 can inhibit the growth of such kinds of tumors as colorectal cancer [17], lung cancer [18], breast cancer [19], pancreatic cancer [20], and acute leukemia [21].

HCC is a highly vascularized tumor, and thus, the antiangiogenesis treatments such as arterializations and embolization have been applied but the overall clinical effect is not satisfying [4, 12]. The destruction of the local HCC blood supply was expected to cause tumor starving and then necrosis, but for the orthotopic HCC *in vivo*, the absence of tumor blood supply also stimulates the endothelial cellular growth directly and indirectly. They stimulate even tinier new tumor vessels to grow up as a compensation. In HCC development, the tumor new vessel formation is an independent risk factor on HCC progression, metastasis, and recurrence. The hypothesis is that Rg3 might increase the HCC tumor-bearing animal survival by inhibiting new tumor vessel formation which has been raised in a rat model of endometriosis [22]. In a few in vivo studies, ginsenoside Rg3 combined with gemcitabine or cisplatin on angiogenesis had been tested on lung cancer in mice [23, 24]; ginsenoside Rg3 combined with metronomic temozolomide had been tested on glioma cancer in rat [25], combined with fiber for inhibiting scar hyperplasia of the skin [26] and prostate stromal cells [27], but the exact molecular mechanism is unclear.

We have previously demonstrated the antitumor potential of ginsenoside Rg3 (Rg3) against HCC *in vitro* and *in vivo* [28]. When applied in Hep1-6 and HepG2 HCC cells, Rg3 induces HCC cell apoptosis via the intrinsic pathway by altering the expression of Bcl-2. The long-term follow-up study had showed that no matter the single use of Rg3 or the combination use with cyclophosphamide (CTX), Rg3 inhibited tumor growth in a subcutaneous HCC model, while its effect on vascular formation is kept unknown. In this study, benefit from a murine orthotopic HCC model in the liver, the function of Rg3 on angiogenesis was investigated and the possible mechanism was explored.

## 2. Materials and Methods

### 2.1. Ginsenoside Rg3

Ginsenoside Rg3 (Lot number HJ20110802-Rg3) was purchased from Hongjiu Biotechnology Co. Ltd. (Dalian, China). The purified Rg3 extract was dissolved, and 10 mg/kg dosage was orally fed to mice.

### 2.2. Cell Lines and Cell Culture

Hep1-6 HCC cells were purchased from the Institute of Shanghai Cell Bank, Academy of Science (Shanghai, China) and multiplied in DMEM (ATCC, Manassas, VA, United States) supplemented with 10% FBS (Shengong, Shanghai, China). The cells were incubated at 37°C in a mixture of 5% CO_2_ and 95% air.

### 2.3. Animal Ethics

Animals received appropriate humane care from a certificated professional staff in compliance with both the Principals of Laboratory Animal Care (NIH publication NO 85-23, revised 1985) and the Guide for the Care and Use of Laboratory Animals approved by the Animal Care and Use Committees of Zhejiang University. All mice were housed in a clean-level animal house in the first affiliated hospital. The mice were caged in 22-24°C, 12 h light/dark cycle, and fed with standard mouse chow and water.

### 2.4. Orthotopic HCC Tumor Model and HCC Animal Model

Female C57BL/6 mice were purchased from Shanghai Experimental Animal Center (Shanghai, China). They were housed to 8-week-old and implanted HCC tumor in an orthotopic manner. Hepa1-6 cells in a log phase were collected and resuspended in 0.2 ml NS. The cells were subcutaneously injected into the back of one C57BL/6 mouse when mice were purchased. After 10 days, tumors were visible and then dissected; the dissected tumors were cut into small pieces immediately (1.0 mm^3^) and then implanted into the liver on the 8-week-old recipient C57BL/6 mouse with percutaneous approach by puncturing a tunnel in the left liver lobe. The anesthesia of mice was performed by intraperitoneal injection of ketamine hydrochloride (100 mg/kg) and atropine (1 mg/kg) (Shanghai No. 1 Biochemical and Pharmaceutical, China).

### 2.5. The Experiment Design and Illustration

**Figure 1:**
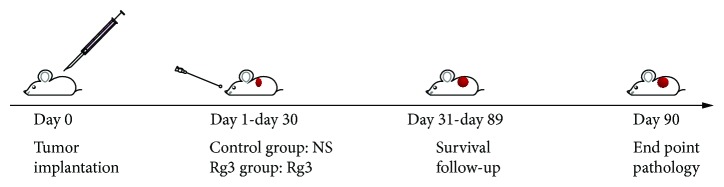
The experiment design and illustration. After implantation with Hep-1 HCC tumor cells (day 0), altogether, 20 HCC tumor-bearing mice were randomly divided into two groups: the control group (*n* = 10) and the Rg3 treatment group (*n* = 10). They were fed orally by normal saline (0.2 ml/mouse, once a day) or Rg3 (10 mg/kg) for 30 days (day 1-day 30). The experiment was investigated for three months.

After orthotopic implantation with HCC tumor (day 0), altogether, 20 HCC tumor-bearing mice were randomly divided into two groups: control group (*n* = 10) and Rg3 treatment group (*n* = 10). They were fed orally by normal saline (0.2 ml/mouse, once a day) or Rg3 (10 mg/kg) for 30 consecutive days (day 1 to day 30). After treatment, the survival study began. The animal technician, who was blind to the study, monitored the mouse weight and tumor size every day. The survival was followed up for three months. Whenever the animal showed the palpable tumor on the abdomen which the preliminary ultrasound study showed, the tumor volume was approximately 2 cm^3^, the animals with the heavy tumor burden were sacrificed by inhaling ether for their welfare according to the animal experiment proposal, and the time was marked as the end. Otherwise, the tumor-bearing mice were kept to the cutoff till the 90th day posttumor implantation. On the 90th day, the end of survival study, when the rest of the animals were all euthanized, the tumor was dissected for weighting, pathology, transmission electron microscope (TEM), and microvessel density (MVD) analysis. The survival difference between two groups was compared by Kaplan-Meier survival analysis. *P* value of less than 0.05 was considered statistically significant. The experiment design is shown in the flow chart in Figure 1.

### 2.6. The Mouse Survival by Kaplan-Meier Survival Analysis

The Hep1-6 HCC tumor-bearing mice were followed up, and the survival time was decided based on the length of time after Hep1-6 HCC tumor was palpable (the volume is about 2 cm^3^). The rate of survival between two groups was compared by Kaplan-Meier survival analysis by SPSS software (version 17.0, SPSS, Chicago, United States).

### 2.7. Tumor Histopathology

When the tumor was as large as 20 mm in diameter, the animal was euthanized and the tumor was dissected and fixed in 40 g/l neutral formaldehyde. After 24 h, it was embedded in paraffin, cut into 3 *μ*m sections, stained with hematoxylin and eosin (HE), and examined under light microscopy.

### 2.8. Apoptotic Cell Identification and Quantification

The quantitative analysis of apoptosis was performed by using pathology and TEM. Apoptotic cells were recognized by the morphological character of condensed nuclear stain with pyknosis and fragmented nuclei. They were quantified as the average of 10 randomly selected fields under a microscope per tumor. All the identification and quantification were performed by software Image Processing and Analysis in Java, (NIH ImageJ, Version 1.42-2).

### 2.9. Tumor MVD

HCC tumor angiogenesis can be quantified by counting the number of new tumor blood vessels, termed as MVD, which is characterized as the highly vascularized area (hot spots) on pathological slides. In this study, the endothelial antibody CD105 was stained by immunohistochemical assays in order to illustrate the active neovascularization in HCC. MVD counting was performed by experienced pathologists with digital camera image acquisition system (Olympus, Japan) and image analysis software ImageJ (NIH, Version 1.42-2, USA). In brief, the hot spots which represent the most vascularized tumor areas were selected under light microscope; then, the exact number of CD105-stained vessels was assessed by the built-in software. The brown color stained CD105-positive cells can be differentiated from the unstained tumor cells, while the preexisting big vessels (>20 *μ*m) were excluded.

### 2.10. Immunohistochemistry

Immunohistochemical staining was performed for CD105 antibody (1 : 50, Dako, CA, USA) in 6 *μ*m paraffin slide. After routine antibody incubation and PBS rinse, the stained sinusoidal spots were captured by Olympus microscope. MVD areas were quantitatively measured using immunopositively stained vessels vs. the total vessels in the field by a pathologist.

### 2.11. Statistical Analysis

Data were presented as mean ± SD. Statistics was performed using SPSS (version 17.0; SPSS Inc., Chicago, IL, USA). Data were analyzed using Student's *t*-test for statistical significance. A *P* value of less than 0.05 was considered statistically significant.

## 3. Results

### 3.1. Rg3 Prolonged the Survival of Hep1-6 HCC Tumor-Bearing Mice

**Figure 2:**
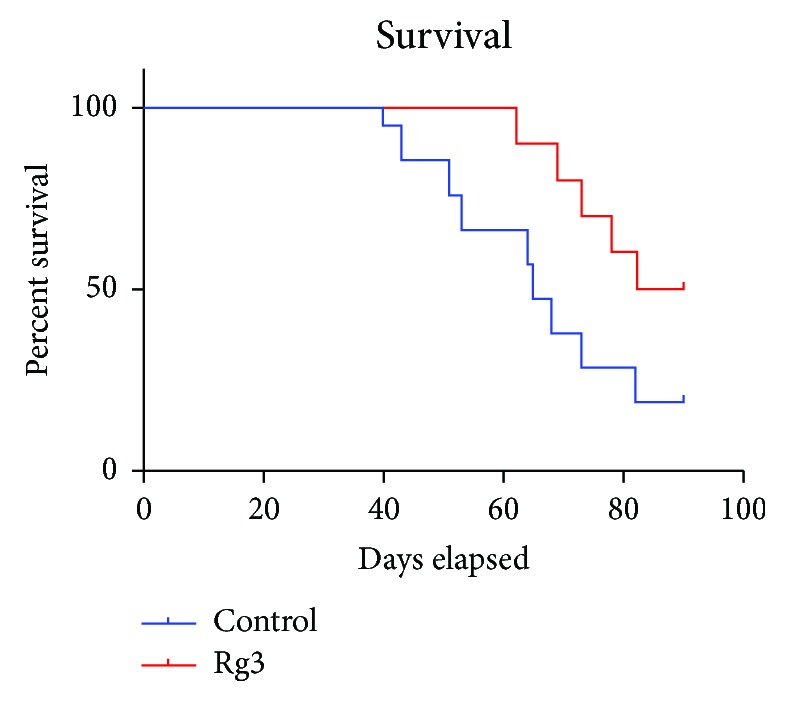
The mouse survival by Kaplan-Meier survival analysis. Hep1-6 HCC tumor-bearing mice were treated with normal saline, control (blue curve), or Rg3 (red curve). Survival was based on the length of time after the Hep1-6 HCC tumor volume reached 2 cm^3^ (time when the animal was euthanized). The median survival for control was 65 days and 86 days for Rg3-treated mice (log-rank test *P* < 0.05). Kaplan-Meier analysis shows that mice with Rg3 treatment had a significant higher survival rate than the control without Rg3 treatment (*P* < 0.05).

The median survival for the control group was 65 days and 86 days for Rg3-treated mice (log-rank test *P* < 0.05). Kaplan-Meier analysis shows that mice with Rg3 treatment had a significantly higher survival rate than control without treatment (*P* < 0.05) (Figure 2). As seen in Figure 2, survival curves of Hep1-6 HCC-bearing mice were treated with normal saline as control (blue curve) or Rg3 (red curve) at a dose of 10 mg/kg. The survival curve indicated that mice with Rg3 treatment had a significantly higher survival rate than control without Rg3 treatment (*P* < 0.05).

### 3.2. The Animal Weight Changed during 90-Day Follow-Up

**Figure 3:**
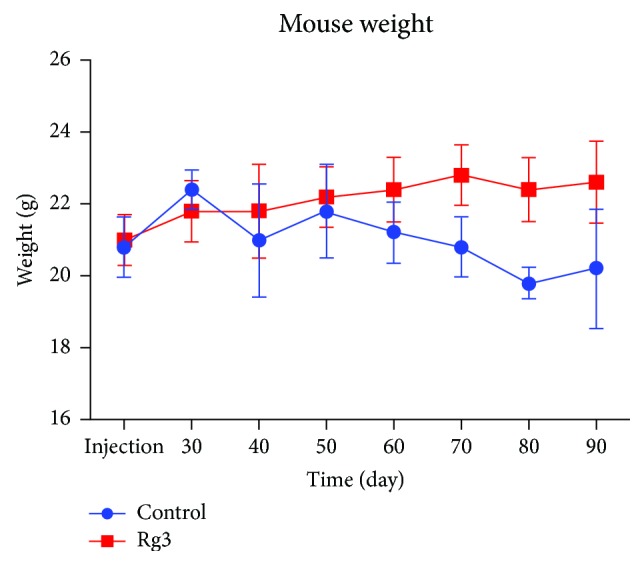
The mouse weight follow-up study. The animal weight in the control and Rg3 groups both increased in the first month after the Hep1-6 tumor cubes were implanted in the liver lobe. After the initial 30 days when the tumor developed, the tumor-bearing mice began to stop gaining weight. Statistical analysis showed that there was no significant difference between the average weights between the two groups, suggesting that the Rg3 had no negative effect on the body weight of the hepatocellular carcinoma-bearing mice.

The mouse weight follow-up study was shown in Figure 3. The twenty newborn mice were divided into two groups, and their weight had no significant difference in the beginning of the experiment. After the tumor implantation on 8-week-old mice, the animals were weighted regularly to check the effect of the HCC tumor and Rg3 on body weight. Both control and Rg3 groups increased their weight in the first month after the Hep1-6 tumor cubes were initially implanted in the liver lobe. After 30 days when the tumor developed, the tumor-bearing mice began to stop the gaining of weight. Particularly in the control group without any treatment, the mouse weight curve even went down the baseline. As a comparison, the body weight kept above the baseline in the Rg3 group. While due to the limited range, the statistical analysis found no significant difference between two groups (*P* < 0.05) suggesting that the Rg3 had no negative toxicity effect on the body weight of the hepatocellular carcinoma-bearing mice.

### 3.3. The Tumor Weight at the End of the Experiment

**Figure 4:**
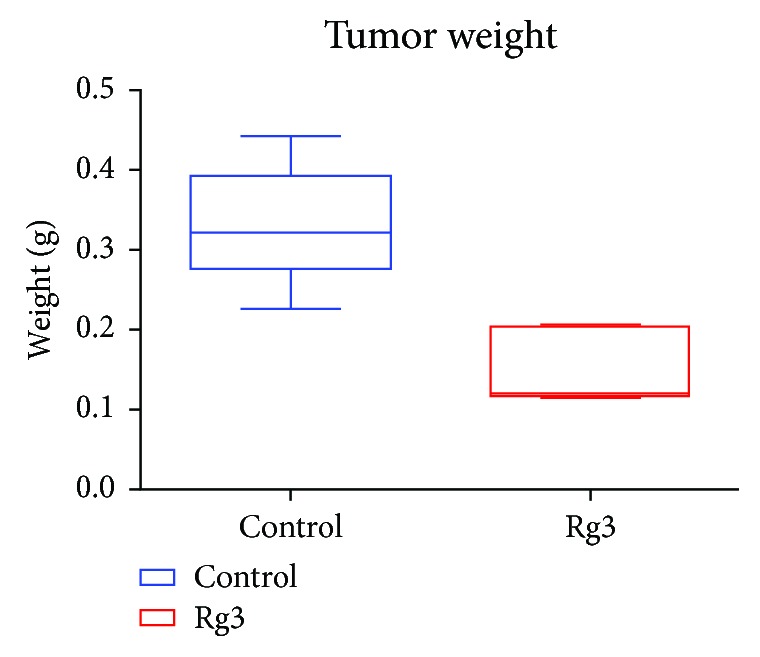
Rg3 inhibit on Hep-1 HCC tumor development *in vivo*. At the end of the experiment, mice were sacrificed to dissect the tumors. The weight of the tumor from mice was scaled. *P* < 0.05 vs. the control group, suggesting Rg3 decreased the Hep1-6 HCC tumor growth in mice.

When the tumor was palpable in the abdomen, which has been confirmed by preliminary ultrasound study that the tumor size reached the upper limit of tumor burden as 2 cm^3^, the tumor-bearing mouse was euthanized by inhaling ether for the animals' welfare. No remote metastasis was found. The tumor lump in the liver was dissected for the weight scaling and further pathological exams. The tumor weight in the Rg3 group was significantly lower than those in the control group (*P* < 0.05), suggesting Rg3 suppressed HCC tumor growth in the liver. The oral feeding with Rg3 significantly reduces HCC tumor growth without obvious poisonous effect (*P* < 0.05) (Figure 4).

### 3.4. Rg3 Induced Apoptosis *In Vivo*

**Figure 5:**
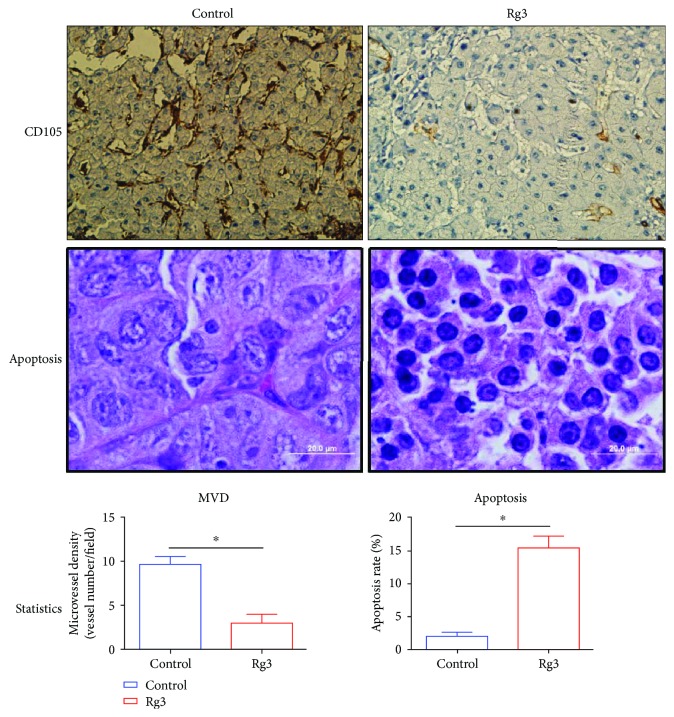
The quantities of apoptotic and microvessel density analysis. Apoptotic cells and microvessels were quantified as the average of 10 fields selected per tumor (magnification was shown as the bar). The CD105 is an endothelium marker that was found highly expressed in HCC. The MVD-CD105 positively stained tumor vessels were significantly less in the Rg3 group than in the control group (*P* < 0.05). In addition, the apoptotic rate increased dramatically in the Rg3 group vs. the control group (*P* < 0.05).

HE-stained pathological sections are shown in Figure 5. Apoptosis was detected in HCC to determine whether Rg3 reduces angiogenesis. Apoptotic cells were identified by pathology. Effect of Rg3 on apoptosis in HCC was identified by pathology. They demonstrate the pathological changes of posttumor implantation. The representative images in the Rg3 group showed the fragmented nuclei indicating apoptotic cells; in the control group, hepatic morphology exhibits the clear hepatic lobular structure and tumor nodules with rich vessels. In the Rg3 group, the tumor clusters showed extensive cell death without infectious neutrophil infiltration. No vessels appeared but there is a loss of endothelial integrity with extensive hepatocellular degeneration in the background.

### 3.5. Rg3 Decreased MVD in HCC

Angiogenesis quantification (MVD) in tumors is performed by counting vessels stained with CD105 (Figure 5). The CD105 is an endothelium marker that was found highly expressed in HCC. The MVD-CD105 positively stained tumor vessels were significantly less in the Rg3 group than in the control group (*P* < 0.05). In addition, the apoptotic rate increased dramatically in the Rg3 group vs. the control group (*P* < 0.05), suggesting that the Rg3 initialized the tumor apoptotic progress, which then weakened the tumor volume and its capability to produce the vascularized network for tumor growth and further metastasis.

## 4. Discussion

Our previous work has shown that Rg3 is a nonpoisonous herb extract that can induce apoptosis [28]. The programmed cell death controlled by caspase protease activation was also found in the in vitro HCC cells and other tumors [23–27]. Apoptosis is cross-linked with multiple pathways associating with tumor development among which angiogenesis is critical for prognosis. In the hypoxia HCC microenvironment, Rg3 inhibited the activity of the implanted tumor cells and weakened the angiogenesis of tumor vascular system as well as the complicated network with immune system and microtumor environment [29–31].

In order to get closer to the clinical features, we assessed the inhibitory effects of Rg3 on tumor growth in an orthotopic murine model. The results showed that there is no difference in body weight found between the Rg3-treated and the control mice. During the first 30 days after the tumor implantation, HCC in both control and Rg3 groups continued to grow progressively, suggesting that the HCC tumor caused nutrition exhaustion whereas the oral administration of Rg3 resulted in a significant reduction in tumor size. At the end of the experiment, tumors from mice in the control group were larger than those in the Rg3 group and were about double the weight of tumors from the Rg3-treated mice, demonstrating that without Rg3 intervention in the early tumor development stage, HCC will grow dramatically, while Rg3 treatment can significantly suppress the tumor growth.

In order to further elucidate the mechanisms of the Rg3 antitumor effects, the cross-link for antiangiogenesis and apoptosis induction effect of Rg3 on orthotopic HCC in vivo was investigated. A marked increase in pyknosis cells was observed in tumor tissue sections and TEM image from mice treated with Rg3 as compared to control, suggesting that Rg3 significantly induced cell apoptosis in HCC; at the same time, the CD105 positively stained tumor vessels were significantly less in the Rg3 group than that in the control group. These data showed that the continuous oral taking of Rg3 decreases neovascularization formation in the early stage of HCC development.

**Figure 6:**
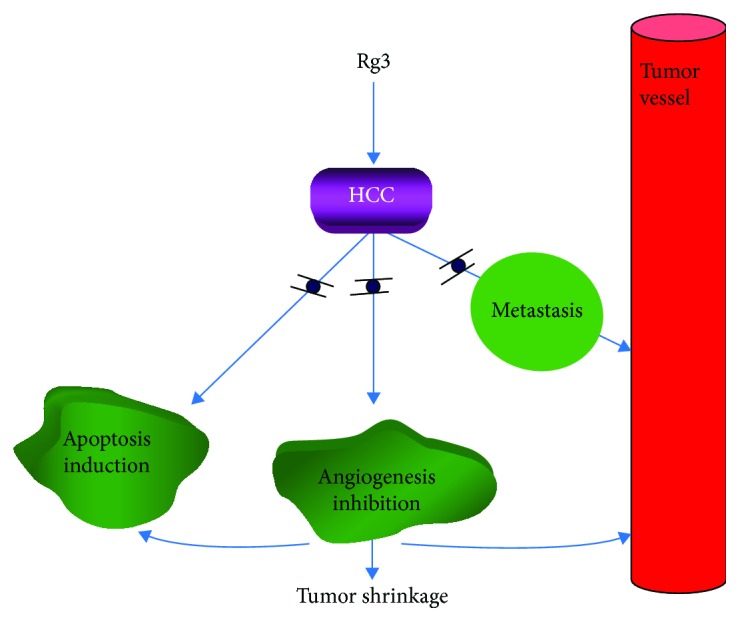
The molecular mechanical illustration. In order to further elucidate the mechanisms of the Rg3 antitumor effects, the orthotopic HCC tumor-bearing mouse was followed up to 3 months with the 30-day oral taking of Rg3 in the early stage of tumor development when the initial tumor was implanted in the liver. Results showed that apoptosis induction and angiogenesis was significantly detected in tumor tissue by Rg3 treatment, contributing to the tumor shrinkage and negative remote metastasis. Rg3 initialized the tumor apoptotic progress, which then weakened the tumor volume and its capability to produce the vascularized network for tumor growth and further metastasis.

As illustrated in Figure 6, in view of the current data, Rg3 showed the potential of antineovascularization in HCC. The oral feeding of Rg3 in the HCC early stage showed the significant blockage of tumor new blood vessel mobilization. As a result, the use of Rg3 in the early stage slows down HCC development and improves the overall survival of the tumor-bearing animals. The current data showed that Rg3 initialized the tumor apoptotic progress, which then weakened the tumor volume and its capability to produce the vascularized network for tumor growth and further metastasis. Our study indicates the clinical potential of using Rg3 in the angiogenesis therapy against HCC.

## Data Availability

The research and result data type used to support the findings of this study are included within the article.
